# Distribution of ilmenite minerals in placer deposits along the middle coast of Southern Brazil using spaceborne and ground-based remote sensing

**DOI:** 10.1371/journal.pone.0314238

**Published:** 2025-02-12

**Authors:** Gabriel Prates Hallal, Carla Cristine Porcher, Bijeesh Kozhikkodan Veettil, Jean Marcel de Almeida Espinoza, Silvia Beatriz Alves Rolim

**Affiliations:** 1 Laboratório de Sensoriamento Remoto Geológico, Centro Estadual de Pesquisas em Sensoriamento Remoto e Meteorologia, Universidade Federal do Rio Grande do Sul, Porto Alegre, Brasil; 2 Centro de Estudos em Petrologia e Geoquímica, Instituto de Geologia, Universidade Federal do Rio Grande do Sul, Porto Alegre, Brasil; 3 Laboratory of Ecology and Environmental Management, Science and Technology Advanced Institute, Van Lang University, Ho Chi Minh City, Vietnam; 4 Faculty of Applied Technology, School of Technology, Van Lang University, Ho Chi Minh City, Vietnam; 5 Laboratório de Física Experimental,Instituto Federal de Educação Ciência e Tecnologia de Santa Catarina, Caçador, Brasil; Bangladesh Council of Scientific and Industrial Research, BANGLADESH

## Abstract

Titanium oxide is of fundamental strategic importance in the global market as it is used as a raw material by several industries, such as medical prostheses, paints, pigments, and, more recently, electronic chips. The main source of titanium oxide is ilmenite, a mineral deposited in many coastal areas of the world, including the state of Rio Grande do Sul in Southern Brazil in its central coastal plain, under specific morphodynamic conditions. Some geological targets, such as mineral oxides, show distinct thermal spectral features. The present study evaluated the surface concentration of ilmenite in Southern Brazil using thermal spectroscopy (μFT-IR). The emissivity spectral signatures of pure ilmenite between 8 and 14 μm were determined and some indicative features were identified. The obtained emissivity spectrum has been employed as a reference for the Spectral Angle Mapper (SAM) and Linear Spectral Unmixing (LSU) image classification algorithms. An image from the Advanced Spaceborne Thermal Emission Radiometer (ASTER) sensor (AST_05 emissivity product) was used to recognize the occurrence and assess the richness of the ilmenite. The outcomes of the present study indicated pixels with ilmenite concentration between 0 and 29.6%, with the highest concentration occurring under the transgressive dune field. In contrast, a lower concentration is found in the backshore. To obtain the degree of purity of the ilmenite, a quantitative microanalysis of the samples was conducted in a scanning electron microscope (SEM), and the results indicated that 80% of the minerals were ilmenite. Qualitative microanalysis showed that ilmenite is in the primary alteration phase, with a low degree of weathering and a lower concentration of impurities. Integrated techniques for analyzing multispectral and hyperspectral data in the thermal infrared were able to identify and map minerals rich in titanium oxide (ilmenite) quickly, effectively, at low cost, and non-destructively.

## 1. Introduction

About 90% of the titanium dioxide (TiO_2_) and titanium metal (Ti) produced worldwide is derived from ilmenite (FeTiO_3_). Due to its ability to form light and resistant metallic alloys and being inert to humans, titanium has applications in different areas, such as prostheses in biomedicine, pigments in manufacturing paints and plastics, the aerospace industry, and nanotechnology [1, 2]. Due to its electrical and magnetic properties, ilmenite behaves like a semiconductor material, which is important in the production of chips in the growing electronics industry [3]. The current semiconductor crisis, amplified by the COVID-19 pandemic, brought negative economic and technological consequences, indicating the significance of developing the worldwide supply chain [4].

Deposits of ilmenite and other heavy minerals of economic interest are found in many coastal regions of the world [5, 6] and are associated with the evolution of coastlines. An example is the placer deposit on the middle coast of the coastal plain of Rio Grande do Sul (PCRS) formed about 5.6 thousand years ago during the last transgressive and regressive sea-level cycles [7]. This deposit has been known since the 1970s. Currently, the Rio Grande Mineração S.A. mining company holds the rights to the mining areas and seeks environmental license from the competent bodies [8].

Orbital sensors with adequate spectral and spatial resolutions make remote sensing a valuable tool in geological studies. The Advanced Spaceborne Thermal Emission and Reflection Radiometer (ASTER) has 14 spectral bands (near, shortwave, and thermal infrared) five of which are in the thermal infrared region (bands 10 to 14), with a spatial resolution of 90 meters. The ASTER is one of the sensors on board the spacecraft Terra, which is the first satellite of the Earth Observing System (EOS) series, aimed at long-term observations of the Earth’s surface, biosphere, atmosphere, and oceans. It flies at an altitude of 705 km in a sun-synchronous polar orbit, crossing the equator at 10:30 h GMT-3, with an average duty cycle of 8% per orbit, and a revisit period of 16 days [9].

The thermal infrared region of the electromagnetic spectrum corresponds to the radiance emitted from surfaces. From the radiance measured in the sensor, the parameters of land surface temperature (LST) and land surface emissivity (LSE) can be recovered from the inversion of Planck’s Law. These parameters are important in remote sensing and geological and environmental studies [10, 11]. Particularly, LSE is a key parameter in the characterization of geological targets, since the main mineral groups such as silicates, sulfates, and some oxides, present spectral diagnostic features in the TIR region [12–15].

The area coverage of an ASTER scene is approximately 60 x 60 km, processed at the L1 level (radiance recorded on the sensor), and stored at the LP-DAAC (Land Process Distributed Active Archive Center). ASTER higher-level L2 products are available on demand via Earth Data Search—NASA, such as AST_05 (LSE) and AST_08 (LST), generated from AST_09T (surface radiance TIR) [9]. The accuracy of these products depends on the accuracy of the atmospheric profiles used. Atmospheric parameters of temperature, water vapor, ozone, and aerosols are inputs to the MODTRAN® radiative transfer model. The AST_05 contains the pixel-by-pixel surface emissivity measurements, produced using the TES (Temperature and Emissivity Separation) algorithm [16, 17]. In geological remote sensing, the AST_05 emissivity product makes it possible to obtain the distribution of a given target (mineral) through hyperspectral image classification techniques practically and accurately [10, 18, 19].

In emission spectroscopy, molecular vibrational movements produce diagnostic spectral features in minerals [20]. Spectroscopy allows the use of reference spectra (endmembers) in comparison with spectra of pixels from orbital sensors [21, 22]. Some minerals, such as silicates and oxides, have a diagnostic feature in the field of TIR [12, 23, 24]. In TIR, the fundamental vibration of the quartz Si-O molecule produces a diagnostic spectral feature, between 8 and 9.5 μm, known as Reststrahlen bands [25, 26]. Ilmenite presents a rectilinear spectrum in the optical domain of the electromagnetic spectrum (VIS-SWIR) allowing it identifiable in the thermal region of the electromagnetic spectrum [27, 28]. Obtaining mineral signatures from the VIS to the TIR regions can be performed in the field, laboratory, or even by accessing available spectral libraries, such as ASTER, JPL (Jet Propulsion Laboratory), and JHU (Johns Hopkins University) [29, 30]. The μFT-IR Model 102F ultraspectral radiometer is used in the field and laboratory for retrieving temperature and emissivity variables from radiance measurements of targets [10, 31, 32]. To perform spectroscopy, it is important to know the composition of the analyzed sample, and for that, scanning electron microscopy is a technique capable of providing information regarding the degree of mineral purity of the sample [33, 34].

The development of orbital sensors and the advancements in image processing/classification algorithms have expanded the science of spectroscopy and geological remote sensing [35]. The Spectral Angle Mapper (SAM) algorithm, even though the spatial resolution of the image is a limitation, is a practical and fast method of mapping by comparing spectra [36, 37]. The Linear Spectral Unmixing (LSU) algorithm is a subpixel analysis model that overcomes such limitations to evaluate the proportion of the target of interest, considering the pixel value as a linear combination of the targets within the pixel [38–40]. Several studies on geological remote sensing have been developed using the SAM and LSU classification algorithms in recent years [5, 35, 41–43]. The current study calculated the surface distribution and concentration of ilmenite in the AST_05 product based on Spectral Angle Mapper (SAM) and Linear Spectral Unmixing (LSU) spectral classification methods and reference spectra (endmember) of ilmenite.

## 2. Methodology

**Fig 1:**
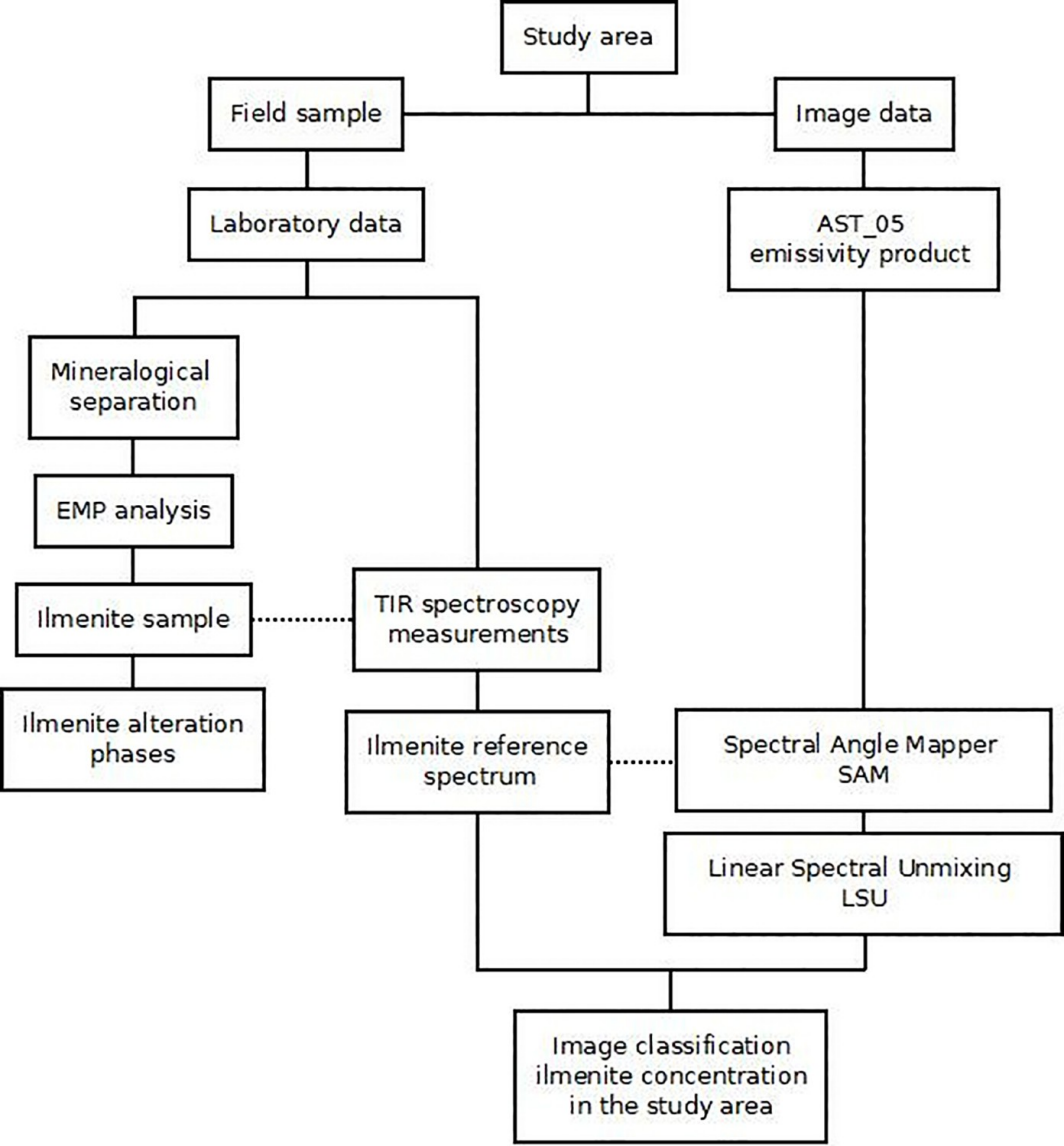
Flowchart of the study.

The spectral signatures (from 8 to 14 μm) of ilmenite were obtained using a portable Fourier Transformed Infrared spectroradiometer (μFT-IR M102F) at the Geological Remote Sensing Laboratory (LabSRGeo) in the Federal University of Rio Grande do Sul (UFRGS), Brazil. In addition, the microscopic analysis of the samples was carried out in a Scanning Electron Microscope (SEM) JEOL JSM-6610LV at Laboratório de Geologia Isotópica (CPGq-IGEO-UFRGS) to estimate the degree of purity of the collected samples. The spectral signatures obtained were used as the reference spectra to identify the mineral target (ilmenite) from the surface emissivity product (AST_05) of the ASTER image, integrating the spaceborne and ground-based remote sensing approaches. The steps implemented are summarized in Fig 1.

### 2.1. Study area and geological settings

**Fig 2:**
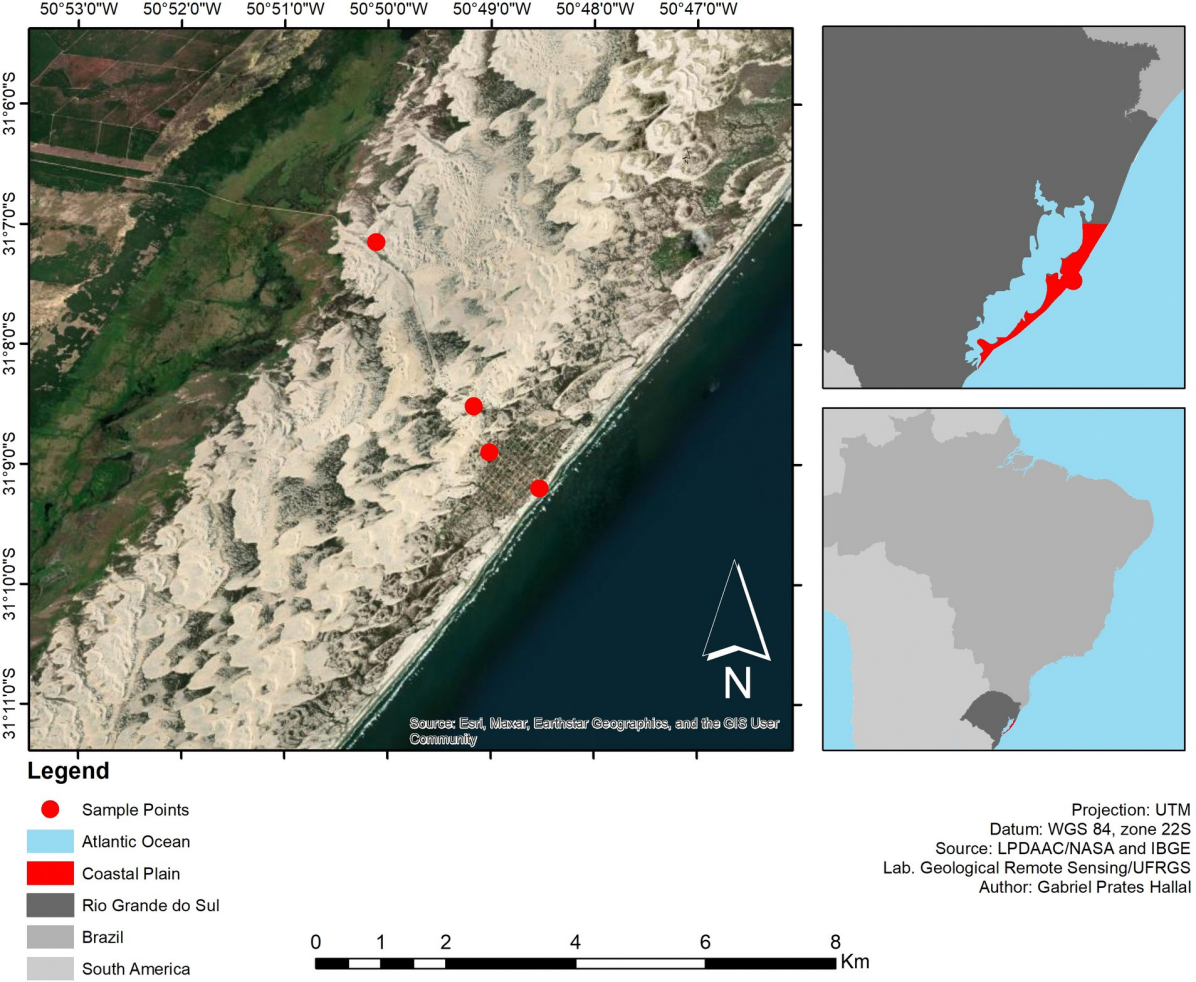
Geographical location of the study area and sample collection points in Southern Brazil.

The city of Mostardas (31°06′25″ S; 50°55′15″ W) is located on the middle coast of the coastal plain of Rio Grande do Sul state. It is limited to the east by the Atlantic Ocean and to the west by Patos Lagoon (**Fig 2**). The coastal plain of Rio Grande do Sul has a NE-SW orientation along 625 km of coastline, with areas having erosion and progradation. Heavy mineral deposits are found on the coast of the Rio Grande do Sul sector where the recessive barrier is dominant, on the extreme south coast near the city of Chuí, on the central coast of the state [7].

The concurrent incidence of recessive barrier and transgressive dunes indicates a link between erosion, formation of transgressive dune fields, and concentration of heavy detrital minerals [44] as it occurs on the central coast, where the resort of Mostardas is located (sample collection site and study area of the present study). The evolution of the lagoon-barrier system, especially barriers III and IV, and the formation of the aeolian placer are related to the last two regressive-transgressive events of mean sea level during the Pleistocene (17.5 thousand years ago) and Holocene (5.6 thousand years ago) through the drowning and reworking of alluvial fan deposits and paleodrainage that cut the current continental shelf [45]. Barrier (IV) is predominantly regressive on the central coast, associated with erosion processes caused by the incidence of waves, storm surges, and the passage of cold fronts coming from the south and southeast, mainly in the autumn and winter months, and migration of dune deposits towards the continent by the northeasterly winds, mainly in the spring and summer months [46]. The deposit extends over barrier IV and anchors on barrier III. The study area is configured as an important mosaic of coastal ecosystems that harbors high biodiversity, classified as a Federal Conservation Unit for Full Protection (Lagoa do Peixe National Park), according to federal laws [47].

### 2.2. Collection and processing of heavy mineral samples

The mineral samples were collected at 4 sampling locations shown in Fig 2 (1. 31°9’12" S-050°48’32" W; 2. 31°8’54" S-050°49’1" W; 3. 31°8’31" S-050°49’10" W; 4. 31°7’9" S-050°50’7" W) in the dune field area, especially the frontal dunes, where the greatest accumulation of heavy minerals occurs. The analyses were carried out at the Center for Studies in Petrology and Geochemistry (CPGq), Institute of Geosciences, UFRGS.

First, the hydraulic separation of light and heavy minerals was carried out with the help of a pan disc. Then the light fraction was discarded (quartz and feldspar, while the heavy fraction after drying in an oven (~180°C / 30 min) was subjected to different amperages in the Frantz® Model L-1 isodynamic magnetic separator for concentration of the heavy mineral species based on their magnetic susceptibilities.

Magnetite and ilmenite are the first minerals separated from the fraction due to their high magnetic powers. These two species are difficult to distinguish from one another due to the similarity of color, texture, and magnetic power. In the present work, an amperage of 0.1 A for magnetite and 0.3 A for ilmenite was used with subsequent microanalysis of the samples in electron microscopy. After that, mineral concentrates were mounted in epoxy resin disks and polished for observation at SEM.

### 2.3. Analysis in scanning electron microscopy

For analyzing the mineral composition of the ilmenite concentrate, slides were made for microanalysis using a scanning electron microscope (SEM) JEOL JSM-6610LV. The SEM provides an image of the atomic density, from the emission and backscattering of an electron beam, a characteristic emission of X-rays. The higher the atomic density, the brighter the grain will appear in the image [48].

From BSE (backscattering electrons) digital images in grayscale and x-ray maps, it is possible to detect the presence of minerals rich in iron (Fe) and titanium (Ti) in the scanned area of the sample. Then, 33 grains of each sample (fractions 0.1 and 0.3 A) were selected for quantitative microanalysis. In the selected grains, a punctual scan was performed to obtain the characteristic X-rays using an EDS detector (energy dispersive spectrometer). A quantitative microanalysis provides the identification of the constituent elements as well as the concentration of these elements. The electron acceleration voltage used was 15 KeV, spot size 55, and working distance 11.2 mm, for a period of 30 s for the EDS analyses. Microanalysis allowed the selection of the most representative magnetic fraction of the mineral ilmenite for thermal spectroscopy analysis later in the laboratory.

The quantitative microanalysis allowed the classification of the ilmenite grains according to the degree of mineral alteration [49]. Primary ilmenites have a low degree of alteration, titanium oxide (TiO_2_) content between ~48–53%, and Ti/(Ti+Fe) ratio ≤ 0.5. As weathering acts on the grain, there is a hydrated ilmenite stage with a TiO_2_ concentration between ~53–60% and a Ti/(Ti+Fe) ratio between 0.5 and 0.6. The continuous process of ilmenite alteration continues with the formation of pseudo-rutile with TiO_2_ content ~60–70% and Ti/(Ti+Fe) ratio between 0.6 and 0.7. Following the alteration process, leucoxene is formed with a TiO_2_ content > 70% and a Ti/Ti+Fe ratio between 0.7 and 0.9. The alteration of ilmenite has as a final product the formation of rutile with TiO_2_ content > 90%. Variability in the TiO_2_ and FeO analyses due to impurities such as Al_2_O_3_ and SiO_2_ was eliminated by multiplying every analysis by 100 / [100—wt% (Al_2_O_3_ + SiO_2_)] [50].

### 2.4. Fourier-transform infrared spectroscopy analysis

After SEM analysis, the ilmenite concentrate sample was measured with a μFT-IR Model 102F spectroradiometer under controlled temperature (25°C) and luminosity in the LabSRGeo in UFRGS. With the use of μFT-IR, it is possible to calculate the emissivity and temperature of the target of interest (ilmenite samples in this case). The calibration of the device and atmospheric correction (removal of downwelling radiance L↓λ) were performed by using a reference gold plate (emissivity = 0.04) [51].

Then the emissivity of the sample was calculated according to Eq 1 given below:

$$\varepsilon =\frac{L\lambda -B\lambda (Ts)}{B\lambda (Ts)-L↓\lambda }$$ (1)

Where, Lλ is the spectral radiance, and Bλ(Ts) refers to the Planck equation given by Eq 2.

$$B\lambda (Ts)=\frac{{C1\lambda }^{-5}}{exp(\frac{C2}{\lambda T})-1}$$ (2)

Where C1 and C2 are constants (C1 = 1.191 x 108 W μm^4^ sr^-1^ m^-2^, C2 = 1.439 x 10^4^ μm K).

The removal of the downwelling radiance L↓λ occurred before and after the sample measurements. The accuracy of the downwelling radiance retrieval determines the accuracy of obtaining the sample’s emissivity and temperature measurements. The equipment was calibrated for forty-five minutes to obtain greater accuracy in emissivity measurements [26].

For the calibration process, distinctive temperatures for the cold blackbody (below the ambient temperature) and the hot blackbody (above the sample temperature) were chosen. The assumed calibration range was 10°C to 40°C. Samples of mineral concentrates were heated in an oven to approximately 60°C. Then the temperature and emissivity measurements of the ilmenite and quartz samples were obtained, generating the reference spectra of these minerals.

### 2.5. ASTER imagery

ASTER sensor data from the AST_05 emissivity product, processing level 2, acquired on demand through NASA’s Earth Data Search was used in the present study. In the AST05 products, the pixel-by-pixel surface emissivity is calculated using the temperature and emissivity separation (TES) method for the thermal infrared bands (5 bands each having 90 m spatial resolution) [16]. The spectral range of ASTER bands used are 8.125–8.475 μm (Band 10), 8.475–8.825 μm (Band 11), 8.925–9.275 μm (Band 12), 10.25–10.95 μm (Band 13), and 10.95–11.65 μm (Band 14), respectively.

The accuracy of the AST_05 products will depend on that of the AST_09T surface radiance product, which uses the MODTRAN® radiative transfer model to remove atmospheric effects [9]. The absolute accuracy of AST_05 is 0.05–0.1, and the relative accuracy is 0.005 (unitless). The date and time of the study area’s image acquisition was on 29/01/2014 at 13:36h GMT-3.

### 2.6. Digital image processing

**Fig 3:**
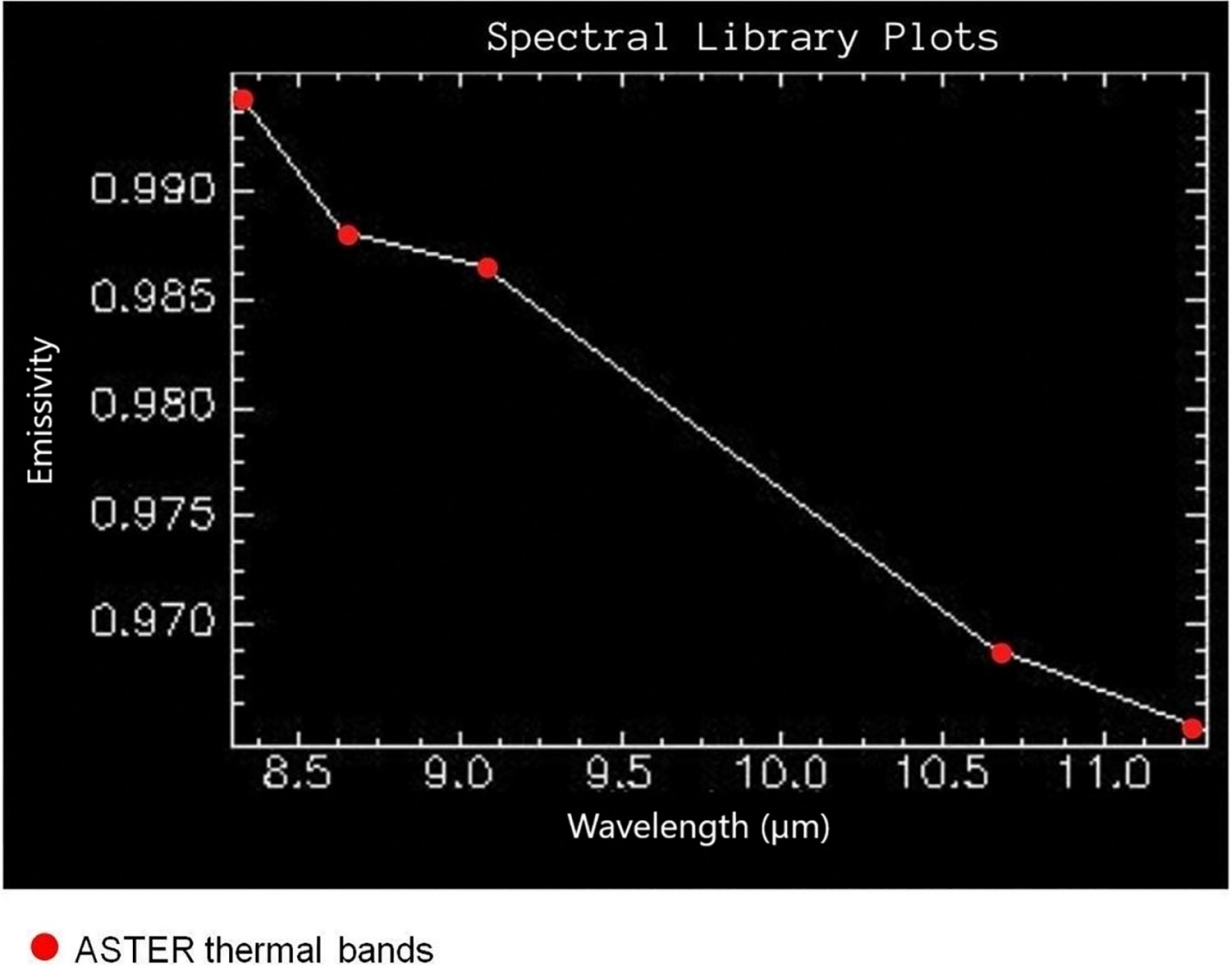
The spectral signature of ilmenite resampled for the thermal bands of the ASTER sensor.

To classify the AST_05 emissivity product image, the Spectral Angle Mapper (SAM) and Linear Spectral Unmixing (LSU) algorithms were used. For this analysis, the emissivity spectrum of the ilmenite sample obtained using μFT-IR Model 102F was used. The signatures were resampled for the response function of the thermal bands of the ASTER sensor (Fig 3). With this, the SAM allows determining or not the presence of a material of interest in the images from the previous knowledge of its spectrum [36, 37] while LSU allows estimating the abundance of ilmenite in pixels previously classified by SAM [38–40].

#### 2.6.1. Spectral Angle Mapper (SAM)

SAM is an algorithm of supervised classification pixel by pixel for digital images that is used to calculate similarities between spectra contained in the library of interest and those contained in the image (endmember vs. pixel). Despite the pioneering works that were developed in the 1990s (e.g. Van der Meer et al. [52]), SAM is still widely being used in geological mapping [53]. Input endmembers can be obtained in the field, in the laboratory, and from spectral libraries (i.e., reference endmembers), or extracted from pixels of the image itself (i.e., image endmembers). To be comparable, however, the spectra must have the same wavelength range. The similarity is calculated by estimating the mean angle of separation (in radians) between spectra pairs (reference endmember *vs*. pixel). The smaller the mean angle (Ɵ), the higher the similarity will be. Spectra are treated as vectors within an n-dimensional space, where n is the number of bands as shown in Eq 3.

$$Ɵ={cos}^{-1}\left(\frac{\sum_{i=1}^{n}m_{i}r_{i}}{{\left(\sum_{i=1}^{n}m_{i}^{2}\right)}^{\frac{1}{2}}{\left(\sum_{i=1}^{n}r_{i}^{2}\right)}^{\frac{1}{2}}}\right)$$ (3)

Where cos^-1^ is the angle between the two spectra.

As the output, the SAM algorithm generates a rule image in grayscale for each endmember, in which digital number (DN) values express the mean separation angle formed between the reference spectrum (endmember) and the spectrum present in the pixel, weighted between 0 and 1. The closer to zero, the greater the similarity and the lighter the pixels. Thus, the output is also a binary image, in which pixels with angle values within a threshold determined by the user will be displayed in white (high correspondence), and outside the threshold (threshold) in black (low correspondence).

#### 2.6.2. Linear Spectral Unmixing (LSU)

LSU is an algorithm that expresses the spectral unmixing analysis model in which the spectrum measured at the pixel is disintegrated into its constituents (fractions, with each fraction referring to each endmember) and abundances at the subpixel level [38–40]. Spectral unmixing models are widely used in geological studies with consistent results with the development of spectral unmixing analysis methods [54]. In LSU, the spectral response of each pixel per band is a linear combination of the spectral responses of its constituents, according to Eq 4.

rk=sk1f1+sk2f2+sknfn+i (4)

Where *rk*is the spectral response of the pixel in the band *k*; *sk* is the spectral response of the endmember in the *k* band; *fk* is the endmember proportion; and *i* = indeterminate portion.

The number of equations for each pixel corresponds to the number of bands in the image. In the LSU model applied to the AST_05 image, the gray level recorded by the pixel is the linear combination of the emissivity of each of the pure targets (endmembers). In the AST_05 multispectral images, the five TIR bands will produce five linear equations (Eq 4) with *n* unknowns (endmembers). Each equation has two restrictions: 1) the value of the fraction must be between 0 and 1, and 2) the sum of the fractions and the indeterminate portion must be equal to 1.

## 3. Results

### 3.1. Electron microscopy analysis

**Fig 4:**
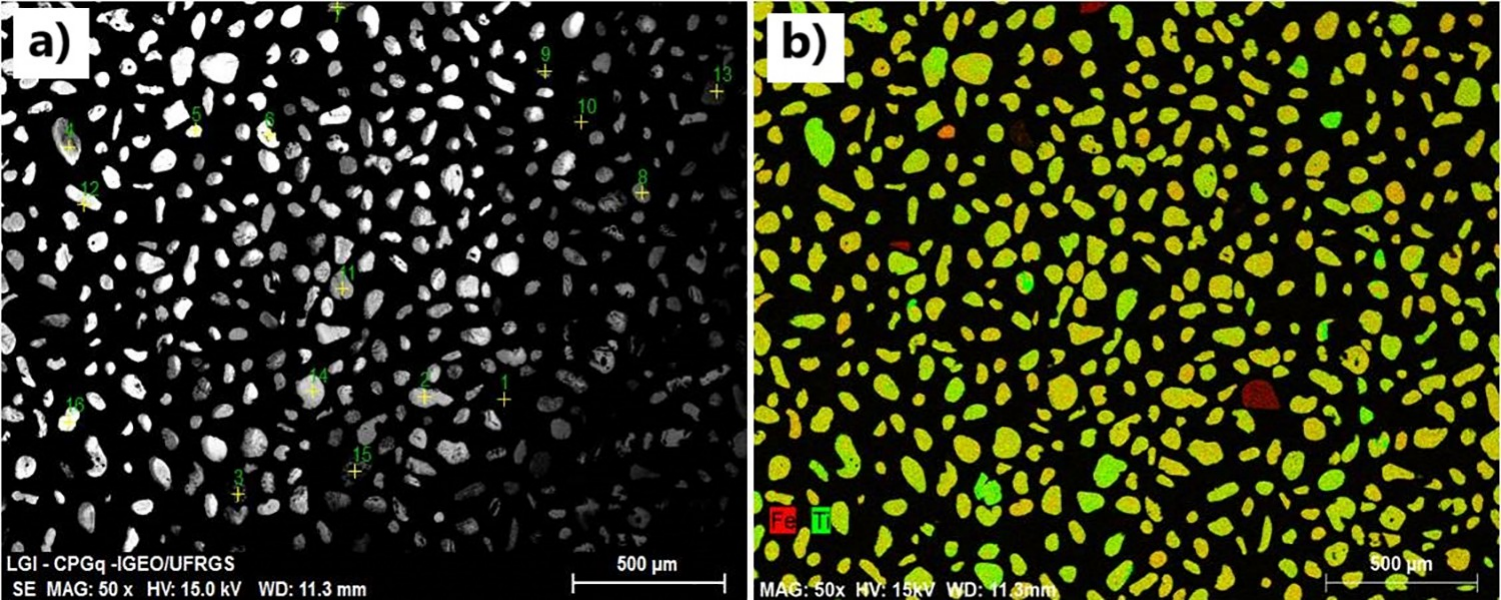
Digital images obtained in electron microscopy: a) the composition spectrum from backscattered electrons (BSE), and b) characteristic X-ray map with an EDS detector based on Hallal et al. [55].

From the processing of the field samples (hydraulic separation between light and heavy minerals, and magnetic separation between heavy minerals) the minerals were concentrated according to the degree of magnetism. Fractions of 0.1 A and 0.3 A were responsible for removing and concentrating magnetite and ilmenite from other heavy minerals. Fractions 0.1 and 0.3 A were analyzed using SEM and digital images were generated from backscattered electrons (BSE), in grayscale (Fig 4A) and characteristic x-ray map in RGB colors (Fig 4B) for qualitative analysis of minerals. The gray shades in the BSE image (Fig 4A) show the compositional contrast related to the atomic density of the grains, which is lighter when the atomic number is higher. The characteristic x-ray map from a previous study by the authors [55] (Fig 4B) collected by the EDS detector indicates the presence of iron (in red) and titanium (in green) in the grains. Based on this, it was possible to verify the prominent abundance of minerals rich in titanium in the scanned area of the sample.

**Fig 5:**
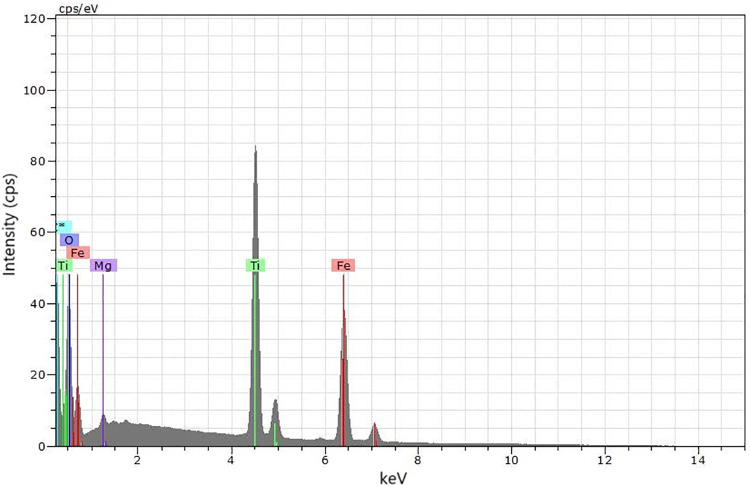
Composition spectrum of primary ilmenite based on microanalysis of the elemental composition of the grains with an EDS sensor.

In the samples, 33 grains of each fraction (0.1 and 0.3 A) were selected to obtain spectra for microanalysis of the elemental composition of the grains (Fig 4) with an EDS sensor. Thus, we obtained the most representative fraction of the mineral ilmenite. Fraction 0.3 A presented 78.8% of ilmenite (Table 1), while fraction 0.1 A presented 45.5% of ilmenite and 42.4% of magnetite. The separation between magnetite and ilmenite was partially achieved by increasing the amperage in the magnetic separator (from 0.1 to 0.3 A), with ilmenite being slightly less magnetic compared to magnetite. Qualitative microanalysis by EDS was performed for 66 grains from samples 0.1 A and 0.3 A. The spectrum below (Fig 5) is an example of ilmenite, composed of 50.6% TiO_2_, 37.9% of FeO, and 2.2% of MgO.

**Table 1:** Mineralogical composition of the 0.3 Ampere fraction.

Mineral	ilmenite	magnetite	tourmaline	zircon	total
**Number of grains**	26	4	2	1	33
**%**	78.8	12.1	6.1	3	100

Ilmenite (FeTiO_3_) undergoes a mineral alteration process that involves the fortification of titanium in the grain as iron is leached from the crystalline structure. Elements such as Mg and Mn replace iron and Al and Si are adsorbed as impurities in the weathering process. This allows the classification of ilmenite according to the TiO_2_ concentration and the ratio between titanium and iron [Ti/(Ti+Fe)]. Primary ilmenite is the nomenclature given to grains with a low degree of alteration, with TiO_2_ between 48–53% and Ti/(Ti+Fe) ratio ≤ 0.5. The spectrum (Fig 5) is of primary ilmenite with TiO_2_ content of 50.6% and Ti/(Ti+Fe) ratio = 0.5.

**Fig 6:**
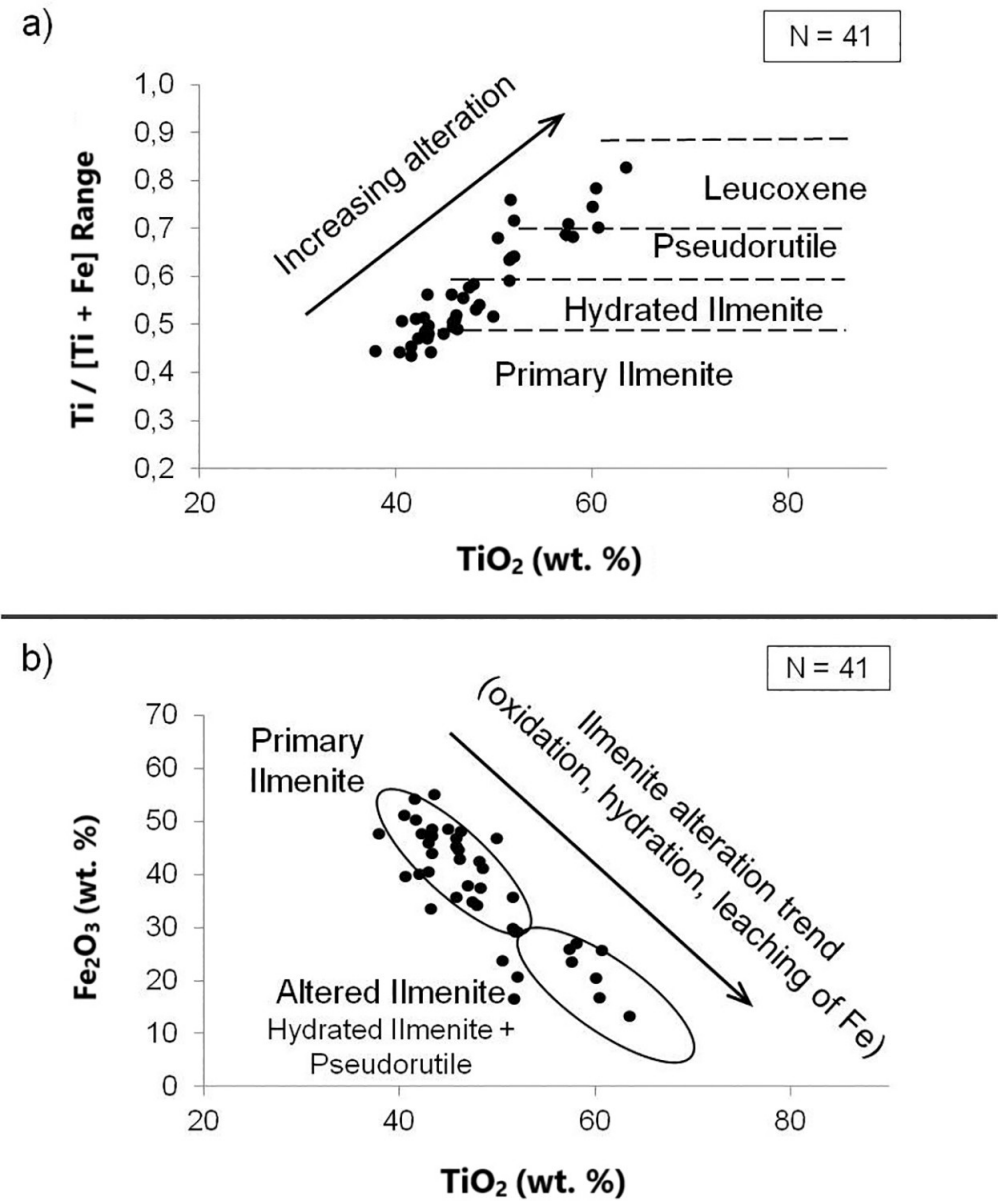
Ilmenite alteration process according to TiO_2_ and FeO contents.

Of the total of 66 microanalyses, 41 ilmenites were identified, and classified according to the degree of alteration, according to the scatter plots below (Fig 6). For the Ti/(Ti+Fe) ratio, the values were between 0.44 and 0.78 (Fig 6A). For the TiO2 content, the grains range from 44.5% to 69.8% (Fig 6B). This shows the continuous process of alteration that occurs in ilmenites during and after deposition, going from primary ilmenite with a low degree of alteration. With a reduction in iron content (Fe^+2^) and an increase in water content in the molecule, the so-called hydrated ilmenite appears as an intermediate phase between primary and pseudorutile. The continuation of the process leads to the transformation of pseudorutile into leucoxene. Transformation into leucoxene raises the impurity content. The average concentration of TiO_2_ in the analyzed ilmenite grains was 53%. The predominant ilmenite class was primary, with 20 spectra (48.8%) identified. Then, the most frequent class was hydrated ilmenite, with 10 grains (24.4%). Then pseudorutile with 7 spectra (17.1%), and finally leucoxene with 4 spectra (9.7%) (Table 2).

**Table 2:** Ilmenite change stage.

Phase	primary	hydrated	pseudorutile	leucoxene	total
**Number of grains**	20	10	7	4	41
**%**	48.8	24.4	17.1	9.7	100

**Fig 7:**
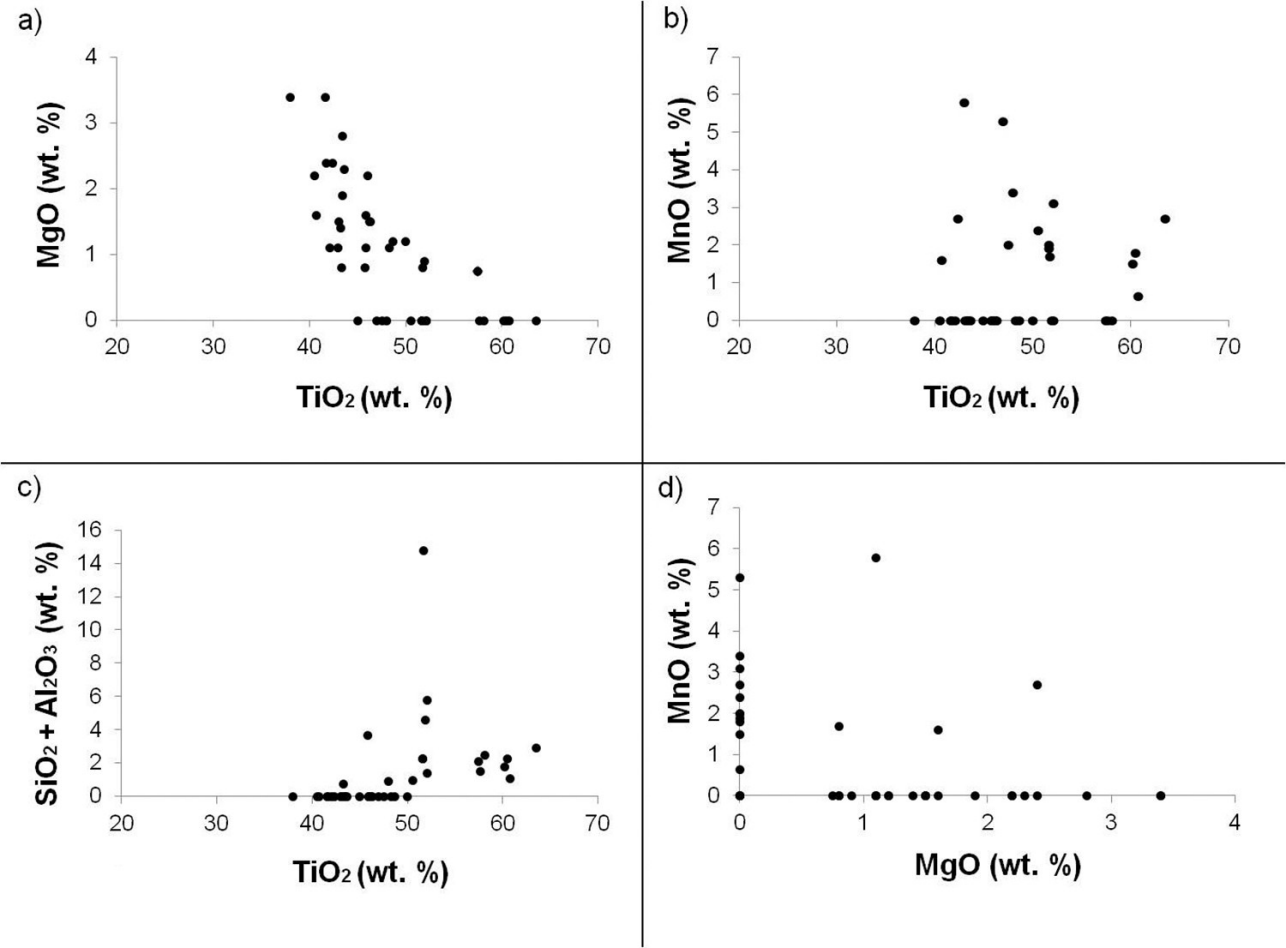
Scatter plots showing the variations in impurity elements with TiO_2_ and MgO contents.

A microanalysis of the main impurity elements found in ilmenite was also performed (Table 3). Scatter plots showed the variation of these elements as the TiO_2_ content and degree of change increased (Fig 7). The concentration of magnesium oxide (MgO) varied between 0 and 3.4%, with an average value of 1.1%. A higher concentration of MgO as a minor element indicates an ilmenite with a lower degree of alteration as shown in the scatter plot (Fig 7A). For the concentration of manganese oxide (MnO), the minerals showed values between 0 and 5.8% (Fig 7B), with an average value of 0.9%. The increase in MnO concentration indicates a stage of alteration in ilmenite as this element replaces leached iron in the crystalline structure. The values found for the concentration of silica (SiO_2_) are between 0 and 6.5%, with an average value of 0.5%. Aluminum oxide (Al_2_O_3_) presented concentration values varied between 0 and 8.3%, with an average value of 0.8% (Fig 7C). Among the 41 ilmenite minerals analyzed, 34 do not contain SiO_2_ and 24 do not contain Al_2_O_3_. In these grains, grains are without the impurity of Al_2_O_3_ and SiO_2_ as there are no occurrences of Al_2_O_3_ and SiO_2_. Iron leaching decreases the volume of the grain, causing micro-fractures through which these impurities are incorporated. The occurrence of MgO and MnO occurs alternately in the ilmenite structure. They occur in place of iron by the similarity of atomic radius, filling the space in the octahedron (Fig 7D). The concentration of the oxides that make up the analyzed ilmenites is summarized in Table 3.

**Table 3:** Quantitative microanalysis of ilmenite grains.

Oxide	Range (%)	Average (%)	Standard Deviation (%)
TiO_2_	38.0–63.6	48.3	6.3
FeO	13.3–51.3	37.5	11.2
MnO	0.0–3.4	1.1	1.0
MgO	0.0–5.8	0.9	1.5
SiO_2_	0.0–6.5	0.5	1.4
Al_2_O_3_	0.0–8.3	0.8	1.3

### 3.2. Emissivity measurements in the laboratory

**Fig 8:**
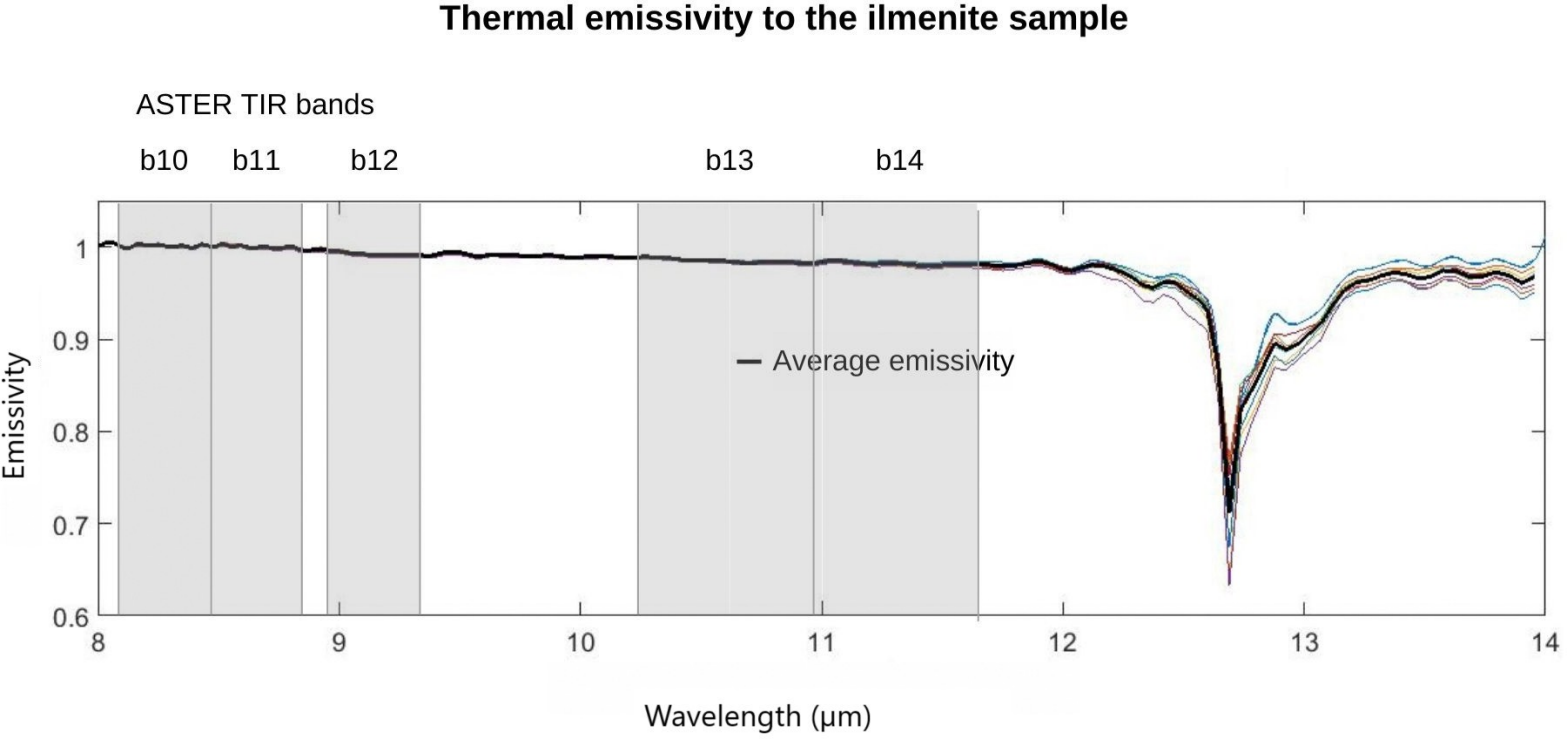
Emissivity measurements for ilmenite sample.

In possession of the 78.8% ilmenite concentrate (0.3 A sample) the emissivity reading of the ilmenite sample was performed. The measurement was carried out using a μFT-IR Model 102F spectroradiometer under controlled temperature conditions (~25°C) and luminosity in LabSRGeo at UFRGS. The spectral curve for the ilmenite sample in the TIR domain (between 8 and 14 μm) shows a maximum emissivity behavior in the first wavelengths (~8–12.5 μm). An absorption peak is observed at approximately 12.7 μm, which is attributed to the vibration of the bonds of the ilmenite molecule (FeTiO_3_). The minimum emission feature is known as Restrahlen [56]. After the most intense absorption peak at 12.7 μm, a second absorption feature of lesser intensity occurs at approximately 12.9 μm, and after returning to emissivity close to 1 from 13.2 μm (Fig 8).

The ilmenite hyperspectral signature was resampled concerning the central value and bandwidth for the response function of the thermal infrared bands of the ASTER data and to be compatible with the bands of the emissivity product image AST_05. In other words, it was resampled in the spectral intervals that correspond to the TIR bands (b10, b11, b12, b13, b14) of the ASTER sensor. The reading values performed by the μFT-IR Model 102F spectroradiometer, which correspond to the beginning and end intervals of a given spectral band, are resampled by the median function, generating a value corresponding to that spectral interval.

### 3.3. Mineral mapping of ilmenite in the coastal plain of Southern Brazil

**Fig 9:**
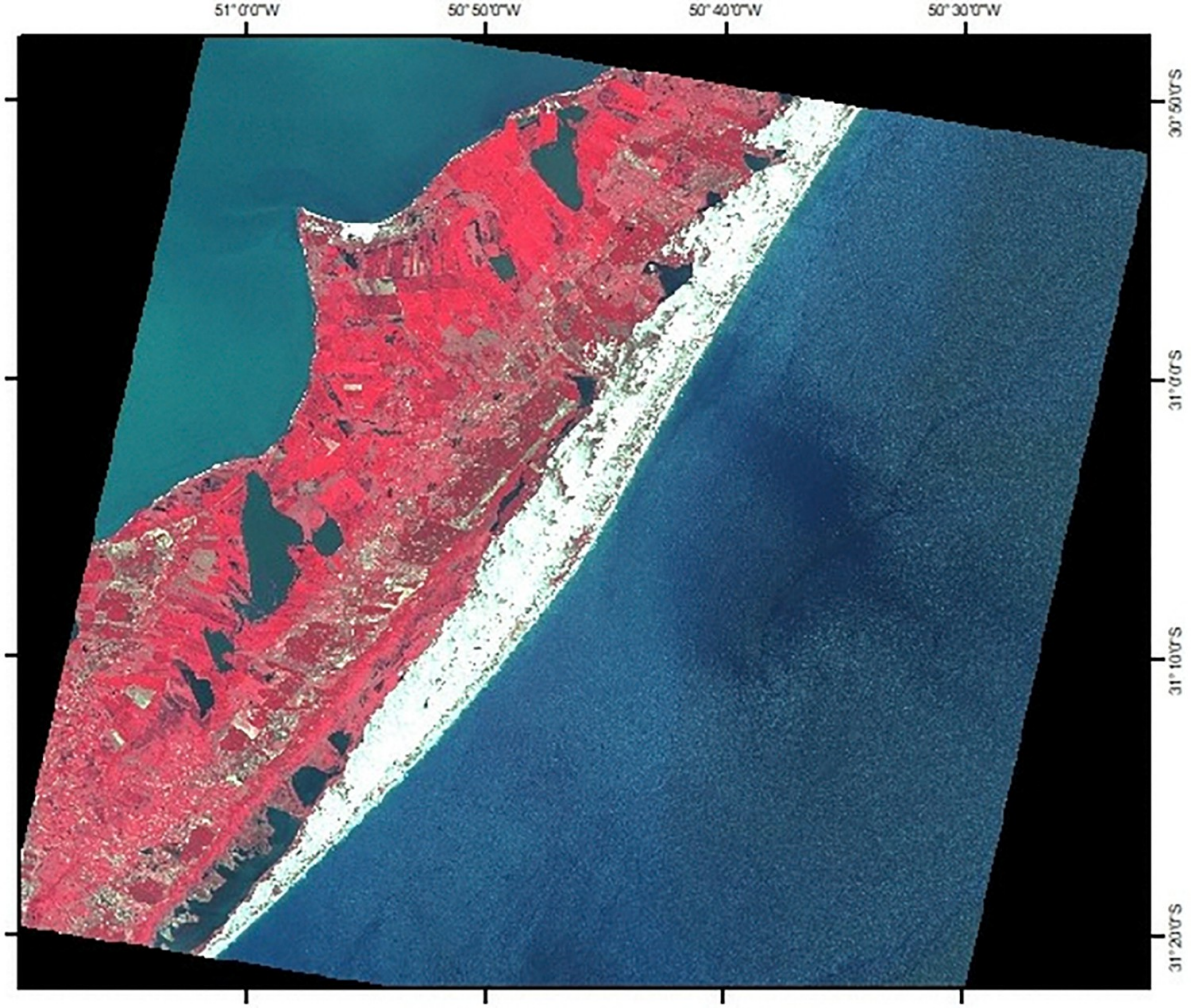
False color composite RGB 3:2:1 ASTER L1B image. Middle coast of the state of Rio Grande do Sul, Brazil.

False color composite (RGB 3:2:1) was performed for the study site, based on the ASTER L1B image. Band 3 (0.78–0.86 μm) of the ASTER sensor comprises the NIR wavelength, where vegetation has a greater spectral response. In the false-color composition performed, band 3 was inserted in the red channel of the RGB, with the vegetation highlighted in red in the image (Fig 9).

**Fig 10:**
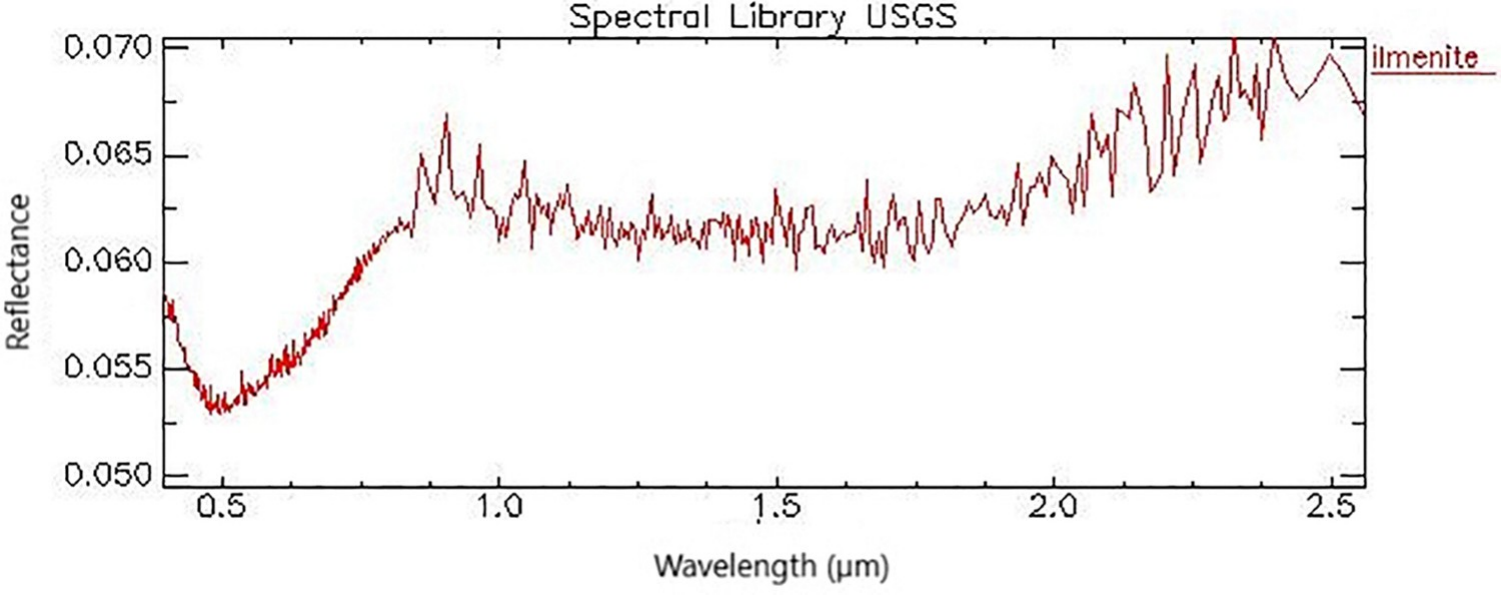
Spectral reflectance signature of ilmenite obtained from the USGS spectral library implemented in ENVI®.

The ilmenite signature obtained in the spectral library version 7 implemented in ENVI® [57], presents a low reflectance between Visible and SWIR wavelengths, with a homogeneous and straight line below 0.1. However, although not very pronounced, the presence of a spectral feature is observed between 0.5 and 1 μm (Fig 10). The presence of this feature indicated the possibility of using VIS and SWIR data for mapping this mineral but limited to the signal-to-noise ratio that can mask the response of this target against other components of the pixel. The ilmenite spectrum, with no significant feature in the VIS and SWIR, increases its confusion with other dark targets of low reflectance, such as organic matter, that occur in the dune field.

**Fig 11:**
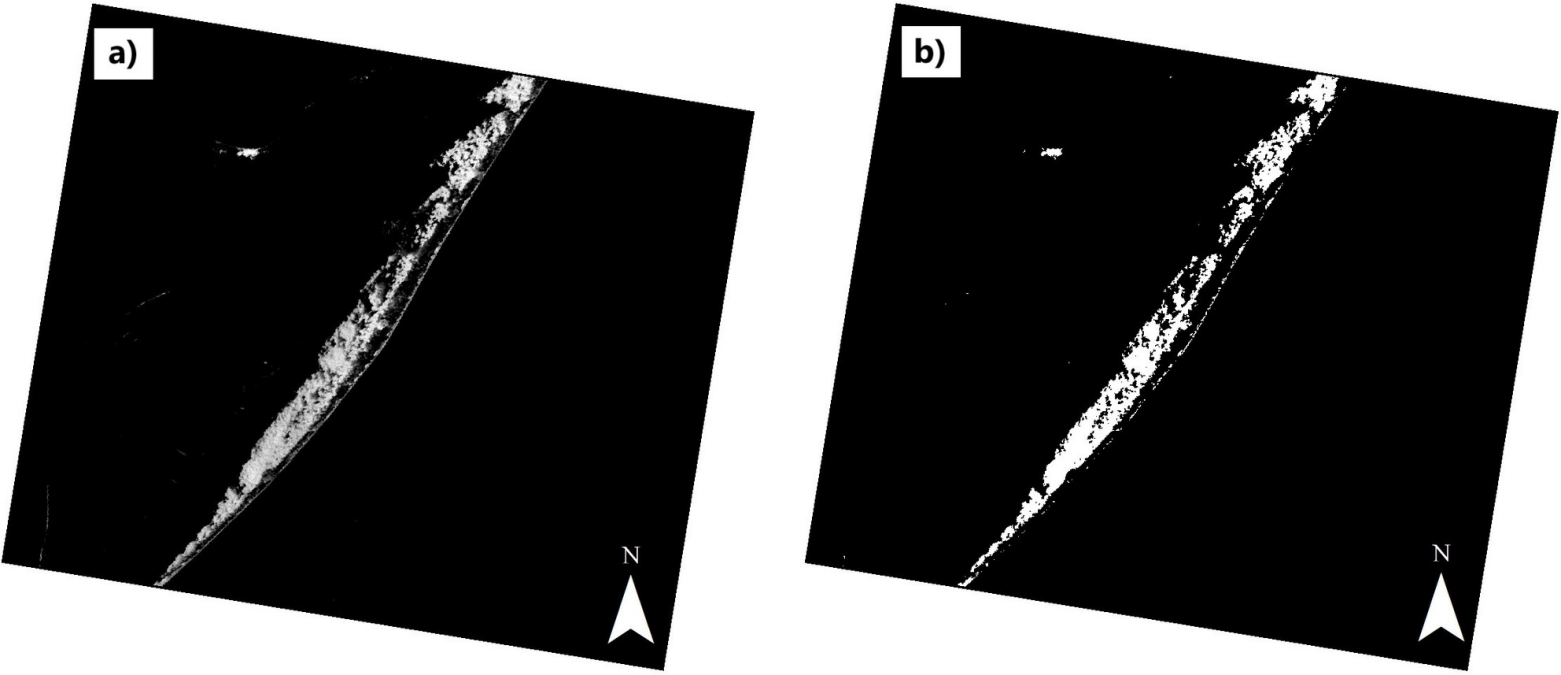
SAM products for ilmenite in the mid-coast of Rio Grande do Sul, Brazil a) rule image b) binary image.

Through the classification of the AST_05 image, the distribution of ilmenite for the area of interest was mapped. This result was sequentially generated using the Spectral Angle Mapper (SAM) and Linear Spectral Unmixing (LSU) algorithms. With this, it was possible to identify the presence of the ilmenite endmember (Fig 11) and to quantify the proportion of this endmember in each pixel of the scene, thus obtaining the distribution and concentration of ilmenite in the dune field of the central coast of the state of Rio Grande do Sul.

As a SAM product, a grayscale rule image is generated, in which the value of the digital numbers of the pixels (weighted between 0 and 1) expresses the angular difference between the spectra reference versus pixel. Lighter pixels with values closer to zero indicate greater similarity between the pair of compared spectra, while dark pixels closer to 1 indicate less correspondence (Fig 11A). In addition, the algorithm employs threshold classification, generating a binary image indicating the presence or absence of the endmember in the respective tested pixel, represented by white (presence) and black (non-presence), as in the image below (Fig 11B). Based on the presence of pixels classified by SAM, areas of interest were filtered for the application of LSU, and the abundance of ilmenite within these pixels was estimated.

**Fig 12:**
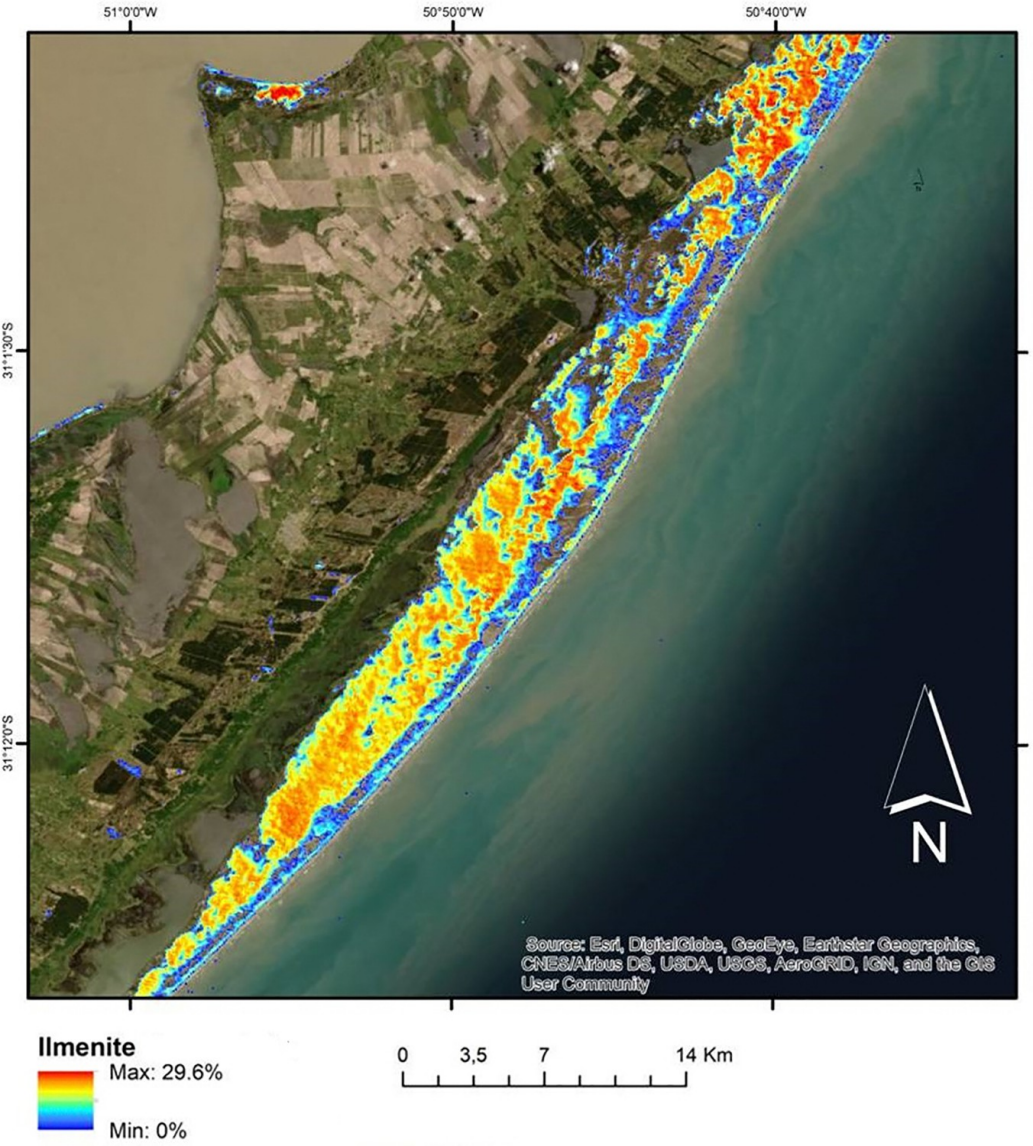
Ilmenite distribution map along the middle coastal plain of the State of Rio Grande do Sul, Brazil.

The estimate of maximum ilmenite concentration for the study area was 29.6% of the sediment, represented by pixels in hot colors, while values of lower concentration of the mineral are represented by pixels in cold colors. The distribution of ilmenite indicates that the field of transgressive dunes on the central coast near the Mostardense resort, concentrates a significant amount of the mineral, with values ranging between about 10% and 25% of the sediment composed of ilmenite (Fig 12).

**Fig 13:**
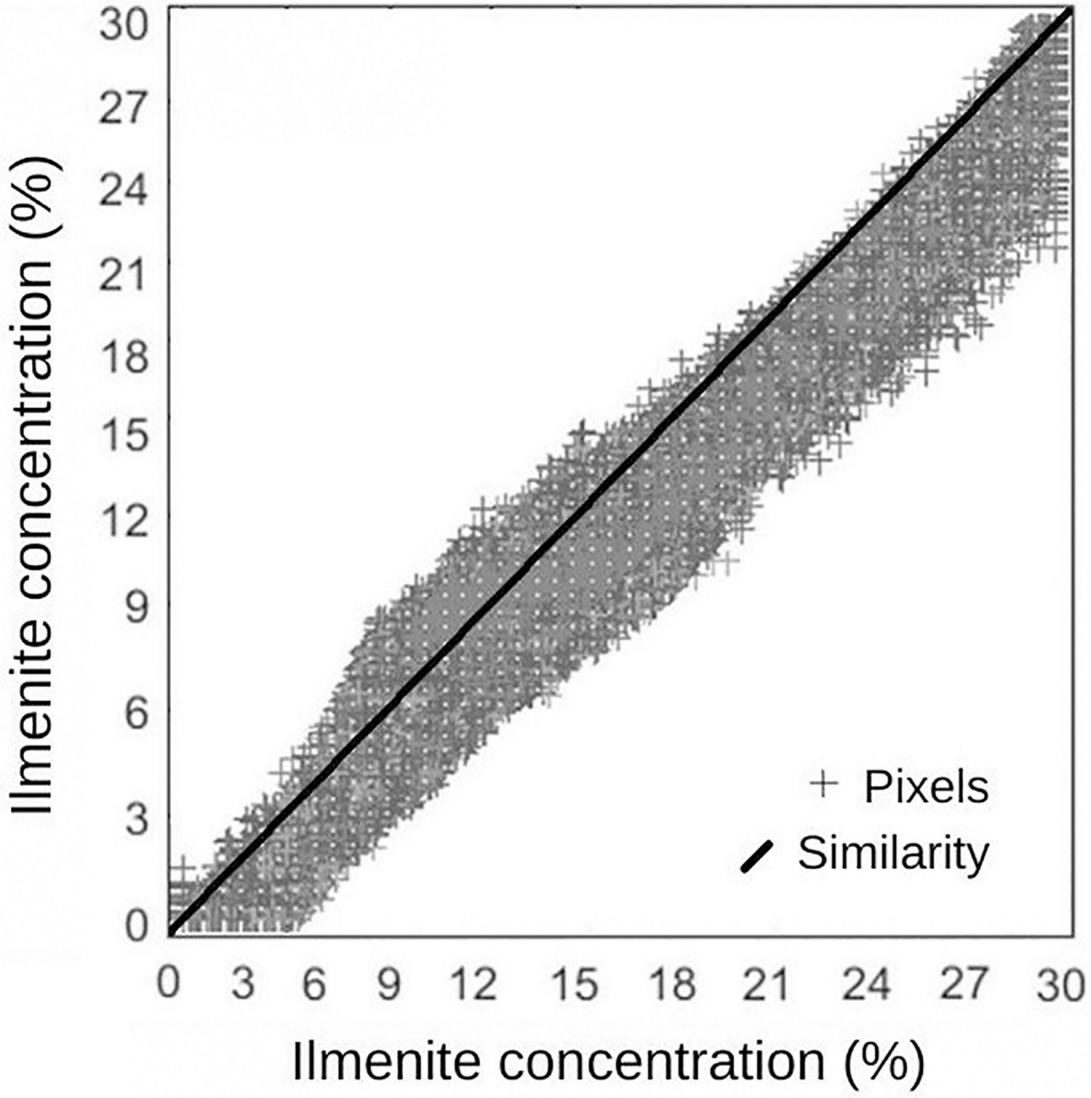
Analysis of the estimated concentration of the presence of ilmenite throughout the pixels of the scene.

The relationship between the spectral signatures of the pixels and the reference signature generated the ilmenite distribution and concentration map. In the graph represented by Fig 13, the distance between a point (representative of a pixel) and the diagonal line indicates the similarity between the spectra (pixel vs. reference). The location of the pixel over the diagonal line would indicate a coincidence between the spectral response of the pixel and the reference. This shows the occurrence of ilmenite in the pixel qualitatively. The ilmenite concentration in the pixel is estimated by the values of the modulus of the vectors, represented on the x and y axes of the graph. The mineral concentration varied between 0 and 29.6%, with the rest of the composition present in the indeterminate pixel (Fig 13).

## 4. Discussion

The focus of this study was to map the surface distribution and concentration of ilmenite for the middle littoral portion of the coastal plain of Rio Grande do Sul. For this purpose, we used Spectral Angle Mapper (SAM) and Linear Spectral Unmixing (LSU) techniques applied to the AST_05 surface emissivity product.

The ilmenite deposit in the study area spreads from the frontal dunes to the transgressive dune field that anchors to the shore of the Lagoa dos Patos. For this dune field, we found ilmenite concentration values of up to 29.6% within the pixel (Fig 12), with the rest being indeterminate (up to 70.4%). The reference spectral signature used in the classification algorithms was obtained in the laboratory with μFT-IR emissivity measurements of the ilmenite sampled from the study area. The signature showed a diagnostic feature at approximately 12.7 μm caused by molecular vibration of the FeTiO_3_ bond (Fig 8).

The ilmenite sample used in the μFT-IR was previously evaluated for purity in electron microscopy analysis. The degree of purity estimated by microanalysis was 78.8% (Table 1). We also performed an initial analysis of the quality of the ilmenite deposit and found a low degree of alteration in these minerals, with a predominance of primary ilmenites (Fig 6), indicating economic potential [50].

### 4.1. Electron microscopic analysis

Scanning electron microscopy and microanalysis of X-ray characteristics in mineral studies allow the identification of the elemental composition, impurities, fracture zones, and pores of the grains [58, 59]. Previous works on the alteration of ilmenite in coastal deposits were carried out in South Africa [60], Brazil [61], Australia [50], and India [62]. Primary ilmenite corresponds to the purest grain, with less weathering and higher market value.

The ilmenite alteration process involves iron oxidation (Fe^2+^) and oxygen incorporation in the formation of pseudorutile (Fe_2_Ti_3_O_9_) [63]. Between the primary ilmenite and pseudorutile phases, an intermediate phase called hydrated ilmenite occurs, with a decrease in Fe2+ content and an increase in water content. The formation of pseudorutile implies a reduction of approximately 6% in the volume of the grain, causing microfractures and porosities. Continuing the alteration leads to the transformation of pseudorutile to leucoxene. The leucoxene phase has higher structural water content and impurities such as Mg^2+^, Mn^2+^, SiO_2_, and Al_2_O_3_. Due to impurities, leucoxene has no market value. In the final stage of alteration, titanium enrichment occurs, while iron is leached and leads to the formation of rutile [64]. Our results obtained from the microanalysis by applying the nomenclature given in Frost et al. [63] indicate the predominance of primary ilmenite grains (Fig 5 and Table 2) with a low degree of weathering (Fig 6).

The characteristic x-ray image (Fig 4B) and x-ray microanalysis of the grains (Fig 5) showed that the magnetic separation between magnetite and ilmenite was possible, with an increase in amperage, from 0.1 to 0.3 A. Because it is more magnetic, the magnetite was retained in the magnet in the first pass at 0.1 A, concentrating the ilmenite in the second pass at 0.3 A (Table 1). The alteration of the ilmenite causes a change in the magnetic susceptibility because of the leaching of iron and the incorporation of impurities, the magnetism decreases [65, 66].

In spectroscopic analysis, the construction of spectral libraries is preceded by electron microscopy to verify the purity of the sample to be measured [20, 27]. The data measured from the collected samples indicated a concentration of 78.8% of ilmenite in the sample (Table 1) with the change of amperage in the magnetic separator. Scanning electron microscopy and x-ray microanalysis allowed us to infer the stage of alteration of the ilmenite and the degree of purity of the sample used in the sequence of the work, with spectroscopy measurements in the laboratory.

### 4.2. Emissivity measurements in the laboratory

TIR spectroscopy has been used for understanding the spectral features of minerals since the 1960s. More recently, Laakso et al. [67] studied the spectral features of rare earth oxides in TIR, and Hecker et al. [21] studied the application of TIR spectroscopy for mapping feldspar by remote sensing. Christensen et al. [27] and Xie et al. [20] highlight the importance of determining the composition and purity of the sample and the descriptive care in the preparation and acquisition of spectra.

It is in the TIR where minerals of silicate groups and some oxides present spectral features [20, 24]. The spectral features result from the selective absorption of photons that depend on the elemental composition and crystalline structure of the mineral [68]. Absorption causes fundamental molecular vibrations, known as the Restrahlen feature, characterized by minimum emissivity values (maximum reflectance) [23, 69]. Christensen et al. [27] measured emissivity spectra of oxides such as ilmenite, hematite, magnetite, and rutile, identifying that the spectral features of these oxides occur at longer wavelengths (above 10 μm).

In the laboratory, under controlled conditions of temperature and luminosity, we took temperature and emissivity measurements of the ilmenite sample using μFT-IR. The spectral signatures we obtained identify the Restrahlen feature at 12.7 μm (Fig 8). Minor impurities, such as those found in the process of alteration of the ilmenite in the study area (Fig 7), do not affect the spectrum of the dominant mineralogy in the TIR. However, they can change the color of the sample and cause variations in the VIS spectrum [12]. Spectral libraries of ASTER, JPL (Jet Propulsion Laboratory), JHU (Johns Hopkins University), and USGS (United States Geological Survey), containing thousands of spectra of mineral species, from Visible to TIR in standard data format, describe the purity, size of the particle, the origin of the sample and the spectrometer provided a quick and practical resource for geological mapping and eliminate the tedious sample collection and laboratory measurement steps [29].

However, in this work, we chose to create the spectral library itself; according to Beddel et al. [70], better results in geological mapping can be expected from specific samples from the study area. To perform measurements with the μFT-IR, we considered the stabilization time and thermal balance of the equipment to avoid noise in the signal and obtain better accuracy, as recommended by Käfer et al. [26], highlighting the quality of high spectral resolution, and the level and portability of μFT-IR for validation of multispectral orbital data in TIR.

Ilmenite is an opaque mineral that does not present a diagnostic spectral feature in the VNIR-SWIR, with rectilinear spectra and low reflectance (Fig 10A) [27]. The opacity of this mineral is due to charge transfers between Fe^2+^, Fe^3+^, O^2-^, and Ti^4+^ ions. Ilmenite has a rhombohedral trigonal crystalline system with the elements iron and titanium alternating in layers of ions (Fe^2+^ and Ti^4+^) bonded to oxygen atoms (MO_6_). In addition to the metal-ligand Ti-O and Fe-O bonds, Ti-Fe intermetallic interaction also occurs [71].

The emission spectroscopy technique applied in our work acquires the spectrum in a similar way to orbital remote sensing data and allows a comparison between data. The spectral library we created for ilmenite served as input data for algorithms SAM and LSU to classify the distribution of this mineral in the placer deposit on the coastal plain of Rio Grande do Sul.

### 4.3. Mapping of ilmenite in the coastal plain of Southern Brazil

For the identification and quantification of the distribution of ilmenite on the dune field surface of the study area, we performed the integration of ground-based remote sensing data (i.e., μFTIR thermal spectroradiometer data) with orbital remote sensing data (i.e., surface imaging data obtained by the ASTER). Similar approaches were employed by Ducart et al. [72] for mapping iron oxides and clay minerals in Serra dos Carajás, northern Brazil, by Abubakar et al. [42] in identifying clay minerals associated with geothermal vents in Nigeria, and by Rejith et al. [73] to map heavy minerals in India.

The use of thermal infrared (wavelength between 8 μm– 14 μm) to investigate targets on the Earth’s surface has increased in recent decades [10]. Because of its spectral resolution with five bands positioned in the TIR, the ASTER sensor is extensively utilized in geological studies [53]. Furthermore, advances in digital processing software and image classification algorithms such as SAM and LSU are capable of generating geological maps with accuracy and agility at the subpixel level using ASTER data [37], as in the work on identification of hydrothermal alteration zones associated with Lead (Pb)–Zinc (Zn) mineralization [41, 74], hydrothermal alteration minerals and the presence of copper deposits in Iran [75], the assemblage of hydrothermal alteration minerals and listvenite formation, in Antarctica [54].

Only a few studies utilized remote sensing and digital image processing for mapping heavy mineral deposits in coastal areas. Among these, Chandrasekar et al. [5] used SAM to map coastal heavy minerals in India, showing the potential of applying hyperspectral remote sensing techniques combined with multispectral imaging in mapping heavy mineral deposits in the coastal zone; Ekanayake et al. [76] used Hyperion hyperspectral imaging data and digital processing to map ilmenite in Sri Lanka; Gazi et al. [77] mapped the presence of heavy minerals off the coast of Bangladesh using SAM and Landsat-8 sensor OLI data; Dhinesh et al. [78] used Landsat-7 ETM+ imagery for mapping paleochannels showing heavy mineral deposits in India; Rejith et al. [73] mapped the occurrence of heavy minerals in Kerala coast (India). Rejith et al. [73] used hyperspectral analysis techniques applied to Landsat multispectral imagery to reduce the dimensionality of the data and SAM classifier to map the occurrence of ilmenite in India.

In the present study, the AST_05 emissivity product image was chosen, which represents a fast and accurate way to obtain the LSE directly. The AST_05 products were validated in studies that used pseudo-invariant targets such as the Algodones Dunes, United States [79, 80], and Cidreira dunes, southern Brazil [19], in addition to the documentation in the sensor manual [9]. Rowan et al. [81] evaluated the AST_05 emissivity data when analyzing the lithological compositions of the Mordor complex, Australia. With reference spectra obtained from the image itself applied to the SAM algorithm; the authors were able to detect the variations in the silica content of the rocks. Aboelkhair et al. [82] identified granitic rocks of the albite type in the desert of Egypt using AST_05 image with emissivity spectra of laboratory samples and the JPL/NASA spectral library for rocks and minerals.

With this approach exposed, we identified the presence of ilmenite in the dune field of the study area by remote sensing using the SAM algorithm (Fig 11) on the AST_05 image and the ilmenite signature obtained by us in the laboratory (endmember) (Fig 8). The SAM classifier algorithm has been successfully applied in geological mapping over the past decades. Girouard et al. [83] applied the SAM method to images from different sensors (i.e., QuickBird and TM/Landsat-5). According to the authors [82], regardless of the low spatial resolution of the Landsat TM (30 m), the sensor was more effective in differentiating rocks due to its higher spectral resolution, with bands positioned in the SWIR region. Chandrasekar et al. [5] analyzed heavy mineral deposits in India, with reflectance data from the ETM/Landsat-7 sensor. Using “pure pixel” spectra obtained from the image and then applied to the SAM algorithm, the authors [5] were able to map the presence of zircon, garnet, and monazite. However, ilmenite did not rank well due to its low reflectance. Honarmand et al. [79] highlighted the potential of using TIR data from the ASTER sensor applied to the SAM algorithm for mapping silicic rocks of hydrothermal alteration. The spectral resolution of the ASTER sensor (specifically bands 10 and 12 in the TIR) detects the diagnostic feature of the Si-O vibration and makes it possible to identify the shift of the feature to longer wavelengths when high silicate composition presents.

To quantify the surface distribution over the dune field and its estimated concentration for ilmenite, we used the LSU model, which involves the acquisition of the reference spectrum, reduced to its characteristic bands (i.e., endmember) and a multispectral image containing the same bands collected pixel-by-pixel to produce an estimate of its proportion in the image [52]. In the model, we inserted the spectrum of the ilmenite sample that we obtained in the laboratory using μFT-IR (Fig 8). The estimated proportion of ilmenite focused on an interval from 0% to 29.6% in the pixels, and most of the pixels presented values between 10 and 15% of ilmenite (Figs 12 and 13).

The LSU is a linear model of spectral mixing that, when considering the endmembers spatially separated within the pixel, generates subpixel images (lower resolution pseudopixels) with the position and proportion of each endmember (fraction images) [84]. Applying a linear model (LSU) to spatially mixed targets, as in the case of a sedimentary deposit of heavy minerals, allowed us to obtain the proportion of the target of interest (i.e., ilmenite), but not its position estimated within the pixel. Abubakar et al. [42] used the SAM and LSU algorithms on SWIR data from the ASTER sensor, proving to be able to identify clay minerals (i.e., kaolinite, illite, monotremalite, and muscovite), hydrothermal alteration products indicators of geothermal systems in Nigeria. Gabr et al. [43] explored the potential of VNIR-SWIR data from the ASTER sensor to detect hydrothermal alteration zones that could be possible indicators of gold mineralization. The authors extracted endmembers of interest from the image itself (i.e., alunite, montmorillonite, kaolinite, goethite, hematite, limonite, and jarosite), applied to the spectral mixture model (LSU) and the SAM algorithm to locate the areas of high probability of gold mineralization. Hosseinjani and Tangestani [85], from ASTER data (from the VNIR and SWIR bands), identified the distribution and sub-pixel quantification of groups of hydrothermal alteration minerals (i.e., kaolinite-sericite, pyrophylite-alunite, and chlorite-calcite-epidote) in southeastern Iran. These groups of minerals are indicators of copper deposits. To map them, the authors [85] used endmembers extracted from the images and the LSU algorithm, which was able to discriminate them with a certainty of 82%. Also, according to the authors, the iterative processes of selection of characteristic bands of the targets of interest are relevant, since the results of the spectral mixture analysis are highly dependent on the selection of the endmembers.

In the present study, we chose the TIR, as the ilmenite response is greater in the thermal domain of the electromagnetic spectrum, with a higher signal-to-noise ratio, significantly increasing the accuracy of the estimates, in addition to reducing methodological steps and the computational cost involved. The present methodology can be used to estimate the purity of heavy mineral deposits in coastal areas. This is important as many coastal areas underwent contamination due to anthropogenic activities [86]. Furthermore, the radioactive risk associated with coastal sand samples, such as those (^226^Ra, ^232^Th, and ^40^K) associated with Egyptian black sand [87], can also be analyzed using remote sensing methods.

According to Dillenburg et al. [7] on C14 dating of the basal layer of peat, the heavy mineral deposit of the middle coast of the coastal plain of Rio Grande do Sul began to form in the last 1000 years and is still in the process of formation. It is believed that these minerals originated from the igneous and metamorphic rocks of the Sul-Riograndense Shield, in which paleochannels of drainage cut the current continental shelf [45]. According to the authors, with the rise in sea level and drowning of the channels, the heavy minerals were reworked and deposited with the evolution of the coastal barrier by the action of waves and winds on the coast. Similar kinds of complex geological formations have been observed in various coastal areas, such as in the Abu Murat area in Egypt [88]. Dhinesh et al. [78] used Landsat ETM+ imagery for mapping paleochannels, and the study was able to identify such geological features associated with the formation of deposits of heavy minerals such as ilmenite, magnetite, garnet, and zircon in a concentration of 10% of the sediment in the coastal zone of southern India.

The need for energy transition as well as reduced CO_2_ emissions, and the increasing demand for titanium-rich minerals consider new placer deposits an opportunity [89]. Magnetic properties of ilmenite are capable of increasing the energy efficiency of reactors powered by oxygen combustion [90]. This includes prospecting for lunar deposits by remote sensing using thermal infrared (20–40 μm), where oxides such as ilmenite show prominent spectral features [91]. Subasinghe et al. [6] highlight global growth in demand for heavy minerals, with forecast changes in global production and increases in ilmenite prices by 2030. The authors also address the importance of the mining industry following the United Nations’ sustainable development goals to overcome ecological and socioeconomic challenges involving mineral exploration. Ahmed et al. [92] proposed a technique for exploring heavy minerals on the coast of Bangladesh in which the excavation follows the angle of incidence of waves, to optimize exploration under areas with the highest concentration of heavy minerals, avoiding greater remobilization of sediment.

Ilmenite is a strategic mineral, with increasing demands, in which few countries hold a large part of the production of this ore. Therefore, it becomes important to discover new sources of heavy minerals and diversify the production chain. In this sense, our work innovates in mapping the ilmenite deposit on the coast of southern Brazil, by combining ground-based and satellite remote sensing techniques in the TIR. The creation of its spectral library obtained from laboratory measurements from local samples allowed the effective mapping of ilmenite. Our results give perspective to the use of TIR in mapping coastal deposits of heavy minerals. Integrating field spectrometer data and satellite remote sensing techniques with digital image processing is a promising tool for mapping heavy mineral (ilmenite) deposits in coastal zones using TIR data from the ASTER sensor.

Our work made a shallow quantification of the distribution of the material, which is important in the segment of the present study, a subsurface analysis with local soundings. The relevance of this note is given by the fact already pointed out by Dillenburg et al. [7] who point to a trend of subsurface accumulation greater than superficial for heavy detrital mineral assemblages in coastal deposits.

## 5. Conclusions

From the outcome of this study, the importance of thermal remote sensing in supporting geological prospecting can be underlined. From the integration of field spectrometry data and ASTER satellite imagery, together with analyses by electronic microscopy and digital image processing, the characterization and spatialization of the ilmenite concentration on the surface of the central coast of the state of Rio Grande do Sul in southern Brazil has been carried out. The laboratory measurements with μFT-IR identified spectral features of ilmenite between 8 and 14 μm. The construction of its own spectral library with ilmenite sampled in the study area, and resampled emissivity values for the thermal bands of the ASTER sensor allowed inferring ilmenite concentration between approximately 0 and 30% per pixel along the dune field.

The AST_05 emissivity product image uses SAM and LSU classification algorithms, dispensing the application of atmospheric correction methods and obtaining the surface emissivity parameter from thermal radiance. Scanning electron microscopy allows considering the ilmenite sample with a satisfactory degree of purity (~80%). An initial analysis of the alteration stage of the ilmenite from the study area was also carried out; minerals with a low degree of weathering were found, indicating the good quality of the deposit. For future works, new spectral laboratory measurements need to be carried out for the mineral magnetite, which together with ilmenite are the main species of heavy minerals in the study area, to expand the minerals of the local spectral library and analyze the behavior of the spectral features. We considered the analysis of the entire assemblage of heavy minerals to characterize the placer deposit on the middle coast of Rio Grande do Sul better.

## Supporting information

S1 File Inclusivity in global research. (DOCX)
